# Dietary Polyphenol Intake Is Associated with Biological Aging, a Novel Predictor of Cardiovascular Disease: Cross-Sectional Findings from the Moli-Sani Study

**DOI:** 10.3390/nu13051701

**Published:** 2021-05-17

**Authors:** Simona Esposito, Alessandro Gialluisi, Simona Costanzo, Augusto Di Castelnuovo, Emilia Ruggiero, Amalia De Curtis, Mariarosaria Persichillo, Chiara Cerletti, Maria Benedetta Donati, Giovanni de Gaetano, Licia Iacoviello, Marialaura Bonaccio, on behalf of the Investigators for the Moli-Sani Study

**Affiliations:** 1 Department of Epidemiology and Prevention, IRCCS Neuromed, via dell’Elettronica, 86077 Pozzilli, Italy; simona.esposito@moli-sani.org (S.E.); alessandro.gialluisi@moli-sani.org (A.G.); simona.costanzo@moli-sani.org (S.C.); emilia.ruggiero@moli-sani.org (E.R.); amalia.decurtis@moli-sani.org (A.D.C.); mariarosaria.persichillo@moli-sani.org (M.P.); chiara.cerletti@moli-sani.org (C.C.); mbdonati@moli-sani.org (M.B.D.); giovanni.degaetano@moli-sani.org (G.d.G.); marialaura.bonaccio@moli-sani.org (M.B.); 2 Mediterranea Cardiocentro, 80122 Napoli, Italy; dicastel@ngi.it; 3 Department of Medicine and Surgery, Research Center in Epidemiology and Preventive Medicine (EPIMED), University of Insubria, 21100 Varese-Como, Italy

**Keywords:** biological aging, dietary polyphenols, cardiovascular disease, bone health

## Abstract

Biological aging, or the discrepancy between biological and chronological age of a subject (Δage), has been associated with a polyphenol-rich Mediterranean diet and represents a new, robust indicator of cardiovascular disease risk. We aimed to disentangle the relationship of dietary polyphenols and total antioxidant capacity with Δage in a cohort of Italians. A cross-sectional analysis was performed on a sub-cohort of 4592 subjects (aged ≥ 35 y; 51.8% women) from the Moli-sani Study (2005–2010). Food intake was recorded by a 188-item food-frequency questionnaire. The polyphenol antioxidant content (PAC)-score was constructed to assess the total dietary content of polyphenols. Total antioxidant capacity was measured in foods by these assays: trolox equivalent antioxidant capacity (TEAC), total radical-trapping antioxidant parameter (TRAP) and ferric reducing-antioxidant power (FRAP). A deep neural network, based on 36 circulating biomarkers, was used to compute biological age and the resulting Δage, which was tested as outcome in multivariable-adjusted linear regressions. Δage was inversely associated with the PAC-score (β = −0.31; 95%CI −0.39, −0.24) but not with total antioxidant capacity of the diet. A diet rich in polyphenols, by positively contributing to deceleration of the biological aging process, may exert beneficial effects on the long-term risk of cardiovascular disease and possibly of bone health.

## 1. Introduction

In the last century, the average life expectancy at birth has increased remarkably, from roughly 45 years in the early 1900s to approximately 80 years today. By 2050, over 21% of the global population will be over 60 years of age and this will lead to an increase in many age-related diseases and disabilities, with tremendous costs for public health systems [1,2,3].

Aging is characterized by a time-dependent functional decline that affects most living organisms [4], and is usually indicated by chronological age (CA) [5].

Recent studies proposed that CA can no longer sufficiently reflect the health status of individuals [6], and biological age (BA), the hypothetical underlying functional age of an organism, has gained increasing attention in recent years [7]. The assessment of BA is based on diverse parameters, e.g., blood biomarkers [8,9], DNA methylation [10], spirometry [11] and structural neuroimaging measures [12]. BA and CA may often differ within an organism and for this reason it is reasonable to compute a difference between BA and CA (hereafter called Δage), which may be positive (suggesting accelerated biological aging) or negative (indicating decelerated biological aging) [7].

Indeed, observational studies showed that subjects with decelerated biological aging (i.e., BA << CA), have lower risk for morbidity, disability and mortality [13,14]. Additionally, recent epidemiological research suggested that Δage may play a role in prediction of all-cause mortality [8,12] and hospitalization for cardiovascular causes [14]. Similarly, biological aging accounted for higher risk of all-cause and cause-specific mortality in non-Caucasian ancestries, e.g., African-Americans [15] and Koreans [16].

Therefore, among individuals with the same CA, a difference in measured BA would reflect differences in decreased or increased risk for age-related cardiovascular disease, disability and death.

Lifestyles such as smoking, physical activity and diet are among environmental factors that possibly influence biological aging [14,17,18].

Prior work showed that a healthy diet, such as a Mediterranean diet [10], is associated with decelerated biological aging [10]. Our group recently reported evidence that subjects with high adherence to Mediterranean diet are on average almost 1 year biologically younger than their CA, as compared to those with low adherence [14].

A Mediterranean diet is traditionally rich in foods that are major sources of polyphenols, naturally occurring bioactive compounds that are the most abundant antioxidants in the diet [19].

In addition to being powerful antioxidants, polyphenols also possess anti-inflammatory properties, and their role in the prevention and treatment of various diseases linked to oxidative stress or inflammation, such as cardiovascular disease, has been extensively reported [20]. Also, polyphenol-rich diet are favourably associated with bone mineral density [21]; in particular, high consumption of extra-virgin olive oil, a major source of polyphenols, leads to lower risk of osteoporosis-related fractures [22].

To date, the association between polyphenol-rich diets and biological aging is not fully elucidated, although some data point to a favourable relationship between consumption of antioxidant-rich foods and some markers of aging, such as leukocyte mitochondrial DNA variations [23] and telomere attrition [23,24,25].

There is also a lack of knowledge on whether the total antioxidant capacity of the diet (TAC), which sums up the free radical scavenging ability of antioxidants in foods [26], is associated with biological aging.

In order to fill this knowledge gap, the present study sought to test the relationship between dietary polyphenols and the total antioxidant capacity of the diet with biological aging, using data from a large cohort of Italian adults.

## 2. Materials and Methods

### 2.1. Study Population

We analysed data from the Moli-sani Study, a large population-based cohort study designed to investigate genetic and environmental risk factors in the onset of cardiovascular, cerebrovascular and tumor diseases [27]. At the baseline survey performed between 2005 and 2010, 24,325 subjects (aged ≥ 35 y) were recruited from city-hall registries of the Molise region. Exclusion criteria were: pregnancy at the time of recruitment, disturbances in understanding or willingness, current poly-traumas or coma, or refusal to sign the informed consent form. The Moli-sani study complied with the Declaration of Helsinki and was granted the approval of the Ethics Committee of the Catholic University, Rome, Italy. Details of the study design are available elsewhere [27].

From the initial 24,325 participants, Δage was calculated in a test set of 4772 subjects, as described below and elsewhere [14]. For the present analysis, we omitted individuals reporting dietary questionnaires judged as unreliable by the interviewers (*n* = 179), and participants with lack of information on diet (*n* = 20). The final analysed sample consisted of 4592 subjects.

### 2.2. Computation of Biological Age

To compute biological age, we used a supervised machine learning algorithm called a deep neural network (DNN), as described in [14]. Briefly, we deployed a DNN for the prediction of BA using 36 circulating biomarkers, recruiting centre and sex as input features, and CA of each participant as a label. Biomarkers used included: lipid biomarkers, including triglycerides, high and low density lipoprotein-cholesterol, lipoprotein a and apolipoprotein A1 and B; markers of glucose metabolism such as glucose, C-peptide and insulin; liver enzymes, aspartate transaminase and alanine aminotransferase; cardiac and vascular markers such as NT-proB-type Natriuretic Peptide and high-sensitivity cardiac troponin I; other hormones such as testosterone and vitamin D; haemostasis markers such as D-Dimer; renal markers (uric acid, albumin, creatinine, cystatin-C); inflammation markers such as high sensitivity C-reactive protein; common haemachrome markers, including red blood cell count and distribution width, haematocrit, haemoglobin levels, mean corpuscular volume, mean corpuscular haemoglobin concentration, total white blood cells, lymphocytes, monocytes, granulocytes, neutrophils, basophils and eosinophils; platelet count, mean platelet volume and platelet distribution width.

The DNN was built in R v3.9 (https://www.r-project.org/) through the Keras package v2.4.0 (https://cran.r-project.org/web/packages/keras/index.html). Briefly, we split the available dataset passing QC (*N* = 23,858) into a random training and test set (80:20 ratio), then trained the algorithm over 1000 epochs in the training set and evaluated the accuracy in the test set. Finally, BA for each participant and the resulting discrepancy with CA was computed (Δage = BA–CA) within the test set (*N* = 4772), which was used as the population of study herein. Details on QC, DNN architecture and performance are reported in [14].

### 2.3. Dietary Assessment

Food intake during the past 12 months was assessed by the EPIC food frequency questionnaire (FFQ) [28] validated and adapted to the Italian population, for a total of 188 food items that were classified into 74 predefined food groups on the basis of similar nutrient characteristics or culinary usage.

Through the use of a specifically designed software [29], frequencies and quantities of each food were linked to Italian food tables [30] to obtain estimates of daily intake of macro- and micro-nutrients and energy, and integrated with the TAC values of several foods representative of the average Italian diet, such as fruits, vegetables, oils, beverages, spices, dried fruits, sweets, cereals, pulses and nuts [31].

Polyphenol antioxidant content (PAC) of the diet was appraised by a PAC-score as in Pounis et al. [32], a holistic approach that took into account the potential synergistic effects of polyphenols and likely lowered the possibility for biased regression estimations deriving from multi-correlation of nutritional data For each dietary source of polyphenols (i.e., vegetables, fruits, beverages and alcoholic beverages, for a total of 23 food groups), the mean content in different classes and subclasses of polyphenols was calculated according to the availability of food composition table data. Using this information and the daily consumption of each food source, the total intakes of seven classes and sub-classes of polyphenols were calculated as follows: flavonols (mg/d), flavones (mg/d), flavanones (mg/d), flavanols (mg/d), anthocyanidins (mg/d), isoflavones (mg/d) and lignans (mg/d). The choice of units presented for polyphenols was done according to the units used in the original source [33].

Deciles of total intakes of each polyphenol class and sub-class were then generated; for all polyphenol components, higher intakes (that is, >Q6) scored positively, while lower intakes (that is, <Q5) received negative scoring. The PAC-score ranged between −28 and 28, and resulted from the sum of the seven components. An increase in the score indicated higher total content of polyphenols in the diet.

TAC was measured in foods via the use of three different assays: the trolox equivalent antioxidant capacity (TEAC, mmol Trolox) assay, measuring the antioxidants’ ability to reduce a radical cation in both lipophilic and hydrophilic conditions; the radical-trapping antioxidant parameter (TRAP, mmol Trolox) and ferric reducing-antioxidant power (FRAP, mmol Fe^2+^) assays, evaluating the chain-breaking antioxidant potential and the reducing power of the sample, respectively [31]. The food frequency questionnaire was specifically validated for assessment of dietary TAC against TRAP, FRAP and TEAC values estimated by a 3-day weighed food record and plasma TEAC and FRAP in a group of healthy Italian adults [34].

### 2.4. Ascertainment of Risk Factors

Information on sociodemographic variables, lifestyles and clinical variables were obtained by interviewer-administered questionnaires.

Participants were considered to have hypertension, hyperlipidaemia or diabetes if they reported having been treated with disease-specific drugs.

Leisure-time physical activity for sport, walking and gardening was self-reported and dichotomized as <30 or ≥30 min/d. Height and weight were measured and body mass index (BMI) was calculated as kg/m^2^, and then grouped into three categories as normal (≤25 kg/m^2^), overweight (>25 and <30 kg/m^2^) or obese (≥30 kg/m^2^). Subjects were classified as never-smokers, current smokers or former smokers (reported not having smoked at all over the previous 12 months or more). Education was based on the highest qualification attained and was categorized as (1) up to lower secondary (approximately ≤8 years of study), (2) upper secondary school (8–13 years of study) and (3) postsecondary education (>13 years of study).

### 2.5. Statistical Analysis

Characteristics of the study population were presented as numbers and percentages, or mean values and standard deviation (SD) for continuous variables.

Multivariable-adjusted linear regression analysis (PROC REG in SAS) was used to estimate the relationship between dietary factors (independent variable) and Δage (dependent variable) and results were expressed as regression coefficients (β) with a 95% confidence interval (95%CI). Distribution of missing values was as follows: educational level (*n* = 1), BMI (*n* = 4), smoking habit (*n* = 5), history of cardiovascular disease (*n* = 69), cancer (*n* = 19), diabetes (*n* = 65), hyperlipidaemia (*n* = 45), hypertension (*n* = 43) and menopausal status (*n* = 4). We used a multiple imputation technique (SAS PROC MI, followed by PROC MIANALYZE) to maximize data availability for all variables, avoid bias introduced by not-at-random missing (MNAR) data patterns and achieve robust results over different simulations (*n* = 10 imputed datasets).

We first tested association of the PAC-score with Δage, and then with each polyphenol subgroup intake. Finally, we tested associations with dietary antioxidant capacity measures (TEAC, TRAP, FRAP). Statistical tests were two-sided, and *p* values of less than 0.05 were considered to indicate statistical significance for the analysis of the PAC-score; for the test of different polyphenol categories and antioxidant capacities, the α threshold underwent Bonferroni correction according to the number of markers tested (α = 0.007 and 0.017, respectively).

Each dietary indicator was scaled by its standard deviation so that regression coefficients indicated the variation in Δage for 1 standard deviation change for each dietary indicator.

Based on previous literature and biological plausibility, two models were fitted: model 1 was adjusted for age, sex and energy intake (Kcal/d); model 2 was further controlled for education, leisure-time physical activity, smoking habit, BMI, CVD, cancer, diabetes, hypertension, hyperlipidaemia, menopausal status, hormone replacement therapy and dietary fibre (g/d).

Analyses were performed separately by sex. Appropriate multiplicative terms for testing interaction were included in the multivariable models to test for a difference of effect of dietary polyphenols/TAC by gender.

Data analyses were generated using SAS/STAT software, version 9.4 (SAS Institute Inc., Cary, NC, USA).

## 3. Results

The analysed population consisted of 2381 women (51.8%) and 2211 men (48.2%) with a mean CA of 55.6 y (±11.7), BA of 54.8 y (±8.6) and Δage of −0.75 (±7.72). The majority of participants were low-educated (53.1%), prevalently never-smokers (50.0%) and overweight (42.1%) (Table 1).

As compared to subjects with lower PAC-score (Q1), those participants reporting higher PAC-score (Q4) had lower Δage, were prevalently men, tended to be well-educated and former smokers and practiced more physical exercise, and the women in Q4 were prevalently under replacement hormonal therapy (6.2%) (Table 1).

Greater intake of polyphenols was also associated with higher antioxidant capacity of the diet and increased fibre and energy intake (Table 1).

In multivariable-adjusted analysis, increased PAC-score was inversely associated with the Δage (β = −0.27; 95%CI −0.52, −0.02). None of the polyphenol classes and sub-classes was associated with Δage at the statistical significance thresholds set (α = 0.007). Similarly, no association of Δage with indices of TAC was observed (Table 2).

**Figure 1:**
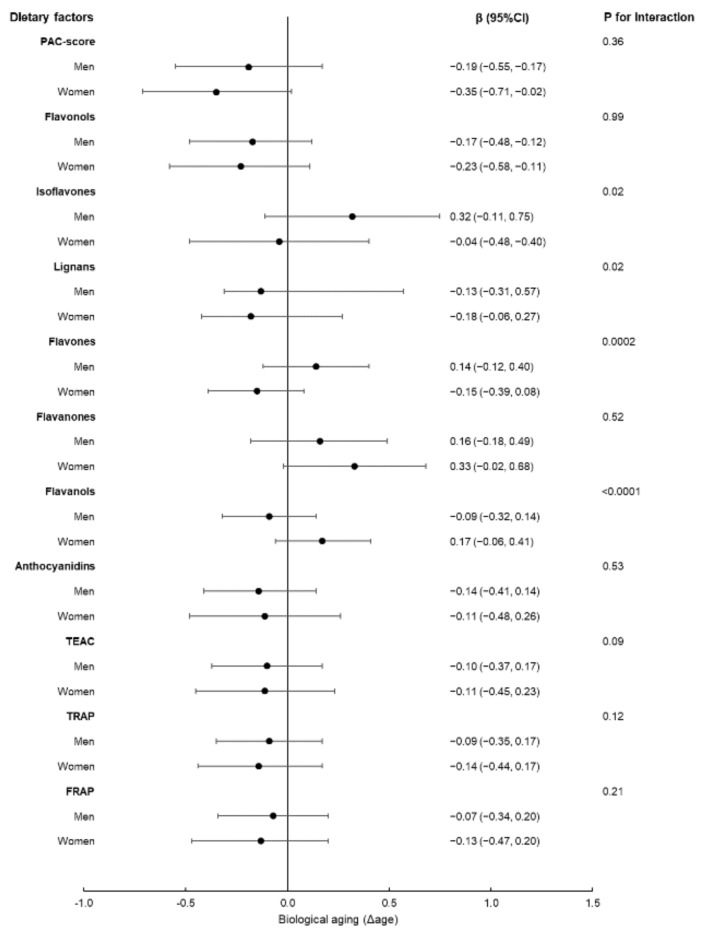
Association of the PAC-score, polyphenol classes and sub-classes and total antioxidant capacity with Δage in men (*N* = 2211) and women (*n* = 2381), by means of regression coefficients (β) with 95% confidence interval (95%CI). Multivariable-adjusted regression model adjusted for age, sex, energy intake, educational level, leisure-time physical activity, smoking habit, BMI, CVD, cancer, diabetes, hypertension and hyperlipidaemia, menopausal status, hormone replacement therapy and dietary fibre (g/d).

Sub-group analyses revealed that the associations of Δage with certain polyphenol sub-classes differed by sex; in particular, isoflavones and flavones were inversely associated with Δage in women but not in men (*p* values for interaction = 0.02 and 0.0002, respectively), whereas lignans showed a greater inverse association with Δage in women as compared to men (*p* value for interaction = 0.02); finally, flavanols were inversely correlated with Δage in men (β = −0.09; 95%CI −0.32, 0.14) while being directly associated among women (β = 0.17; 95%CI −0.06, 0.41; *p* for interaction < 0.0001) (Figure 1).

## 4. Discussion

In a large cohort of Italian adults, a diet rich in polyphenols was associated with decelerated biological aging, an emerging predictor of cardiovascular disease risk. Total antioxidant capacity of the diet was not associated with biological age acceleration.

Our results were in line with and extended prior observations from the same cohort, highlighting an inverse association between biological aging and a high adherence to Mediterranean diet which is abundant in polyphenol-rich foods [14].

Consistently, data from other population-based cohorts previously showed a favourable association between polyphenol-rich diets with various markers of aging, including telomere length [25], cognitive decline [35] and DNA methylation [36], that are susceptible to the antioxidant and anti-inflammatory effects of plant-based diets.

Polyphenols are naturally occurring antioxidants mainly present in a large variety of edible plants (i.e., vegetables, cereals, legumes, fruits, nuts, etc.) and plant-derived beverages such as coffee, tea, beer and red wine [19].

Several epidemiological studies have examined the association between intake of certain polyphenol subgroups and their sources, and the incidence of cardiovascular disease [37] and mortality [38]. Moreover, they reported improvements in inflammatory markers [39] and lipid profile associated with increasing polyphenol intake [40].

The health advantages of fruits and vegetables, rich in polyphenols and antioxidants, have been extensively described also in the regulation of inflammation, a major factor contributing to the senescence—and the reduction of—oxidative stress, which plays a key role in the aging process [41,42,43], and possibly in the etiopathogenesis of osteoporosis [44].

Other studies have also emphasized the impact of diets rich in fruits and vegetables on mitochondrial DNA alterations, implicated in premature aging [23]. Additionally, the benefits of plant-based diets have been studied also with reference to telomere attrition, which is possibly accelerated by oxidative stress and inflammation [25].

Dietary polyphenols likely act on human health through several mechanisms; it has been demonstrated that some polyphenols and their metabolites exert anti-atherosclerotic effects, improve endothelial function and antioxidant status, increase nitric oxide release, and modulate inflammation and lipid metabolism [5,26,31,45,46,47].

The anti-aging properties of dietary polyphenols have been ascribed at least in part to their antioxidant properties that, through several mechanisms including ROS-scavenging actions, contribute to reduce oxidative stress. These mechanisms play an important role in the aging process and various aging-associated chronic diseases [48], including bone mineral density, by preventing oxidation-induced damage to bone cells [49].

Aging is also the result of an imbalance between inflammatory and anti-inflammatory networks [50], a condition often named inflammaging, characterized by elevated levels of blood inflammatory markers that lead to high susceptibility to chronic morbidity, disability, frailty, and premature death [51].

Previous data from the Moli-sani cohort showed an inverse association between dietary polyphenols and a score of low-grade inflammation, which supports the anti-inflammatory action of these bioactive compounds [38].

A consistent body of evidence shows a link between dietary intake of antioxidant vitamins and antioxidant-rich foods and lower risk of mortality [52] and cardiovascular disease [53].

As people eat foods in combination, the evaluation of the total antioxidant capacity of the diet is a more valuable approach to measure their joint antioxidant effects [54]. Also, levels of single antioxidants in food do not necessarily reflect their total antioxidant capacity. Rather, this depends on the synergic and redox interactions among the different molecules present in the food [54].

Diets with high antioxidant potential are inversely associated with frailty [55] and with longer telomeres [56], although they were found to be unrelated to risk of major neurological diseases [57].

Of interest, in our study, the absence of an association between total antioxidant capacity of the diet with decelerated biological aging suggests that the observed inverse association of dietary polyphenols with biological aging may not be exclusively due to their antioxidant potential.

Also, because of the relatively poor bioavailability of many of these compounds, their health promoting effects could not be easily explained by the antioxidant action, which may occur only at high circulating and tissue concentrations [58].

Consistently, analyses in the PREDIMED cohort found no association between the antioxidant capacity of the diet and mortality [59], while observing a reduced risk of CVD associated with dietary polyphenols [37]. Similarly, in a cohort of postmenopausal women, dietary polyphenol intake—but not the dietary total antioxidant capacity—was inversely related to cardiovascular disease risk [60], suggesting that mechanisms other than antioxidant activity may be responsible for the health benefits of polyphenols, including positive modulation of gut microbiota [61] and reduction of inflammation [62].

Despite highlighting a relationship between the total content of polyphenols in the diet with decelerated biological aging, we failed to observe an association with the intake of specific polyphenol classes and sub-classes. This could possibly reinforce the actual usefulness of a holistic approach as implemented through the PAC-score.

Some sex-related differences were observed in our study. In particular, we found that dietary polyphenols and some polyphenol sub-classes were more strongly associated with decelerated biological aging among women than in men.

Such sex-based differences in the relation between polyphenols in the diet and health have not been extensively documented [63]. However, a few epidemiological studies revealed that polyphenol intake is more likely to be associated with health advantages among women than in men, as in a recent study showing that polyphenol consumption was significantly associated with individual components of the metabolic syndrome only in women [64]. Similarly, a positive association of total antioxidant capacity of the diet with pulmonary function was observed among women only, being stronger in premenopausal/never smoker women [31]. These sex-related differences may be attributed to physiological differences between men and women, e.g., body composition, gastrointestinal characteristics, renal function and metabolism, that impact on pharmacokinetic parameters [65]. Additionally, a role for sex hormones might explain the differences between men and women [66] in the association of polyphenols and biological aging. Further studies—possibly analysing interactions with sex hormone titers—are warranted to investigate this hypothesis.

### Strengths and Limitations

This study has several strengths, including a large sample size with thorough measurement of diet and risk factors to minimize confounding. We used an innovative, machine-learning-based measure of biological aging; a deep neural network was applied to a large number of circulating biomarkers, an approach which has, to date, been developed in just a few cohorts worldwide [8,67].

However, there are some limitations that need to be carefully considered. First, the observational nature of the study could not fully rule out residual or unmeasured confounding. In addition, the present findings were derived from a cross-sectional analysis, with consequent difficulty to define directionality of effect. Second, dietary data were based on self-reported information collected through the use of an FFQ and therefore may be susceptible to misreporting that could have led to measurement error and bias or to difficulties in assessing portion sizes and subsequent inadequacies in food composition tables. However, such potential limitations were mitigated by energy adjustment. Also, the validated FFQ used in this study has been previously largely employed and has been reported to have predictive validity for a number of health outcomes [61,62]. Third, our data were obtained from a cohort of adults residing in a small Southern Italian region, and this might limit the generalizability of our findings, although the main characteristics of our population are comparable to those of the Italian Cardiovascular Epidemiological Observatory and are therefore representative of at least the Italian population [68]. Finally, other assessments of biological aging might show a higher accuracy than blood age, e.g., brain age [10], but these are based on expensive instrumental assessments (i.e., Magnetic Resonance Imaging) and not easily applicable to large population-based cohorts.

## 5. Conclusions

Results from this Mediterranean cohort indicate that a diet rich in polyphenols is associated with decelerated biological aging, a novel predictor of cardiovascular disease risk, possibly through mechanisms that go beyond their antioxidant activity.

To the best of our knowledge, this is the first population-based study addressing the association between polyphenol-rich diets and biological aging as reflected by a machine-learning-based measure of blood-based biological age.

Our results have added to the existing literature by expanding knowledge on the anti-aging properties of dietary polyphenols. Future longitudinal analyses are warranted to confirm our results and to test whether a favourable modulation of biological aging is likely to mediate the well-established relationship between polyphenol-rich diets and risk of cardiovascular disease—and possibly of bone health.

## Figures and Tables

**Table 1:** Main characteristics of the study population (*n* = 4952) by quartiles of PAC-score.

		PAC-Score (Quartiles)				
	All	Q1	Q2	Q3	Q4	*p* Value
*N* of subjects	4592	1169	1109	1122	1192	-
PAC-score (mean (SD))	0.77 (13.2)	−16.2 (5.2)	−4.0 (2.5)	5.2 (2.8)	17.7 (4.7)	<0.0001
PAC-score (min, max)	−28, 28	−28.0, −9.0	−8.0, 0	1.0, 10.0	11.0, 28.0	<0.0001
Chronological age (y; mean (SD))	55.6 (11.7)	55.9 (12.4)	56.0 (11.8)	55.0 (11.3)	55.4 (11.1)	<0.0001
Biological age (y; mean (SD))	54.8 (8.6)	55.5 (9.0)	55.0 (8.6)	54.5 (8.3)	54.2 (8.4)	<0.0001
Δage (y; mean (SD))	−0.75 (7.72)	−0.4 (7.8)	−1.0 (7.7)	−0.5 (7.7)	−1.2 (7.6)	<0.0001
Men	2211 (48.2)	42.0	46.9	49.3	54.3	<0.0001
Education						0.06
Up to lower school	2437 (53.1)	56.0	52.7	52.9	50.7	
Upper secondary	1604 (34.9)	33.4	34.8	34.8	36.7	
Postsecondary education	551 (12.0)	10.6	12.5	12.3	12.6	
Unascertained	1 (0.1)					
Smoking status						0.0002
Never smoker	2294 (50.0)	53.5	48.0	49.9	48.4	
Current smokers	1032 (22.5)	24.7	22.4	22.7	20.0	
Former smoker	1266 (27.6)	21.8	29.6	27.4	31.5	
Unascertained	5 (0.1)					
Body mass index						0.01
Normal weight (≤25 kg/m^2^)	1217 (26.5)	28.5	27.2	27.6	22.8	
Overweight (25–30 kg/m^2^)	1932 (42.1)	41.5	41.2	41.8	43.7	
Obese (≥30 kg/m^2^)	1443 (31.4)	30.0	31.6	30.6	33.5	
Unascertained	4 (0.1)					
Leisure-time physical activity						<0.0001
<30 min/d	1589 (34.6)	40.4	36.8	31.4	30.0	
≥30 min/d	3003 (65.4)	59.6	63.2	68.6	70.0	
Cardiovascular disease						0.76
No	4270 (93.0)	93.0	92.0	93.6	93.4	
Yes	253 (5.5)	5.1	6.3	5.5	5.1	
Unascertained	69 (1.5)	1.9	1.7	0.9	1.5	
Cancer						0.96
No	4425 (96.4)	95.9	96.3	96.9	96.4	
Yes	148 (3.2)	3.4	3.2	2.8	3.5	
Unascertained	19 (0.4)	0.7	0.5	0.4	0.1	
Diabetes						0.42
No	4305 (93.8)	94.3	94.2	92.7	93.8	
Yes	222 (4.8)	4.5	4.3	5.6	4.9	
Unascertained	65 (1.4)	1.2	1.4	1.7	1.3	
Hypertension						0.12
No	3232 (70.4)	69.7	69.2	70.3	72.2	
Yes	1317 (28.7)	29.4	29.9	28.8	26.7	
Unascertained	43 (0.9)	0.9	0.9	0.9	1.1	
Hyperlipidaemia						0.74
No	4187 (91.2)	92.1	89.6	91.9	91.0	
Yes	360 (7.8)	7.0	9.2	7.1	8.0	
Unascertained	45 (1.0)	0.9	1.2	1.0	0.9	
Menopausal status						0.95
No	965 (40.5)	41.0	39.7	41.8	40.1	
Yes	1410 (59.2)	59.0	60.3	58.2	60.0	
Unascertained	6 (0.3)					
Hormone replacement therapy						0.02
No	2246 (94.3)	96.7	94.0	92.7	93.8	
Yes	135 (5.7)	3.3	6.0	7.0	6.2	
Polyphenol classes/sub-classes (mg/d)						
Flavonols (mean (SD))	19.3 (10.4)	10.3 (4.2)	15.5 (4.8)	20.1 (5.9)	31.0 (10.8)	<0.0001
Isoflavones (mean (SD))	25.2 (11.4)	14.5 (4.4)	20.8 (3.8)	26.3 (4.9)	38.8 (11.3)	<0.0001
Lignans (mean (SD))	89.4 (42.2)	49.2 (14.0)	72.3 (12.3)	92.6 (16.0)	141.8 (41.1)	<0.0001
Flavones (mean (SD))	0.8 (0.5)	0.5 (0.3)	0.7 (0.4)	0.9 (0.4)	1.2 (0.7)	<0.0001
Flavanones (mean (SD))	35.1 (17.0)	19.5 (7.1)	29.0 (6.5)	37.4 (9.6)	54.1 (17.3)	<0.0001
Flavanols (mg/d; mean (SD))	73.6 (83.6)	38.0 (70.3)	56.5 (52.0)	81.5 (70.5)	116.9 (106.9)	<0.0001
Anthocyanidins (mg/d; mean (SD))	170.2 (104.4)	82.4 (42.8)	135.3 (57.8)	176.8 (67.2)	282.7 (107.0)	<0.0001
Total antioxidant capacity						
FRAP	19.0 (8.6)	15.3 (7.2)	17.9 (7.7)	19.9 (8.2)	22.9 (9.3)	<0.0001
TEAC	6.3 (2.9)	4.9 (2.3)	5.9 (2.5)	6.6 (2.7)	7.7 (3.1)	<0.0001
TRAP	9.2 (4.4)	7.6 (3.8)	8.8 (4.0)	9.6 (4.3)	10.9 (4.8)	<0.0001
Dietary fiber (g/d)	20.6 (7.0)	14.6 (3.6)	18.3 (3.6)	21.2 (4.1)	28.2 (7.0)	<0.0001
Energy intake (kcal/d)	2113.4 (634.9)	1773.3 (512.9)	1998.3 (513.8)	2181.1 (561.3)	2490.5 (693.8)	<0.0001

Values are reported as percentages unless otherwise specified. FRAP = ferric-reducing antioxidant power (mmol Fe^2+^). TEAC = trolox equivalent antioxidant capacity (mmol Trolox). TRAP = radical-trapping antioxidant parameter (mmol Trolox).

**Table 2:** Association of the PAC-score, polyphenol classes and sub-classes and total antioxidant capacity with Δage, by means of regression coefficients (β) with 95% confidence interval (95%CI).

	Biological Aging (Δage)			
Dietary Factors	β (95%CI) ^1^	*p* Value	β (95%CI) ^2^	*p* Value
PAC-score	−0.30 (−0.49, −0.12)	0.001	−0.27 (−0.52, −0.02)	0.03
Polyphenol classes/sub-classes (mg/d)				
*Flavonols*	−0.28 (−0.47, −0.10)	0.003	−0.21 (−0.44, 0.01)	0.06
*Isoflavones*	−0.12 (−0.30, 0.06)	0.18	0.13 (−0.17, 0.44)	0.40
*Lignans*	−0.17 (−0.35, 0.01)	0.06	−0.02 (−0.34, 0.29)	0.89
*Flavones*	−0.02 (−0.19, 0.15)	0.84	−0.02 (−0.19, 0.16)	0.84
*Flavanones*	−0.02 (−0.20, 0.16)	0.82	0.23 (−0.01, 0.47)	0.06
*Flavanols*	−0.04 (−0.22, 0.13)	0.60	0.02 (−0.14, 0.19)	0.78
*Anthocyanidins*	−0.25 (−0.43, −0.09)	0.05	−0.15 (−0.37, 0.07)	0.17
Total antioxidant capacity				
FRAP	−0.01 (−0.21, 0.20)	0.93	−0.13 (−0.34, 0.07)	0.20
TEAC	−0.03 (−0.24, 0.17)	0.74	−0.14 (−0.34, 0.07)	0.19
TRAP	−0.01 (−0.20, 0.18)	0.94	−0.14 (−0.34, 0.05)	0.16

^1^ Regression coefficients (95%CI) obtained from a model controlled for age, sex and energy intake. ^2^ Regression coefficients (95%CI) obtained from a model controlled for age, sex, energy intake, body mass index, cardiovascular disease, cancer, diabetes, educational level, hormone replacement therapy, hyperlipidaemia, hypertension, leisure-time, menopausal status, physical activity, smoking habit and dietary fiber (g/d). FRAP = ferric-reducing antioxidant power (mmol Fe^2+^). TEAC = trolox equivalent antioxidant capacity (mmol Trolox). TRAP = radical-trapping antioxidant parameter (mmol Trolox).

## Data Availability

The data sets analysed in the current study are not publicly available because of restricted access, but further information about the data sets is available from the corresponding author on reasonable request.
